# Supplementary material for: A Likelihood Approach for Real-Time Calibration of Stochastic Compartmental Epidemic Models
Source: PLoS Comput Biol. 2017 Jan 17;13(1):e1005257. doi: 10.1371/journal.pcbi.1005257 (PMC5240920; doi:10.1371/journal.pcbi.1005257)
Supplement: S1 File — (TAR.GZ) [file pcbi.1005257.s014.tar.gz › HSPH_Online-SI-Revision/Additions/EnKF08/n100-extreme_noisy/n100-extreme_noisy_EnKF_table-8.pdf]

1	R0	{2.76052, 16.3913}	{2.05701, 4.37331}	{{1.01088, 2.64898}, {3., 4.91371}}	{32.2514, 73.3194}	inf	695.	16 329.4	{6806.21, 56 490.7}	2250.54	1.20404	newly	289.367	16 360.3	{6592., 55 507.}	5553.83	1.55808	Reff	2.73137	0.297933	{0.0683934, 0.464532}	89.0922	ar	92 090.	171 855.	{105 877., 273 593.}	91.0052
2	R0	{2.18202, 15.1734}	{2.19191, 8.43857}	{{0.406095, 2.93112}, {3., 13.1599}}	{44.4049, 44.3858}	inf	2011.	4999.34	{2894.99, 4414.39}	148.6	0.247712	newly	1160.	3976.62	{1226., 6229.}	242.812	0.344531	Reff	2.10076	0.42384	{0.0725865, 0.67538}	79.8245	ar	84 264.	192 259.	{181 868., 232 959.}	128.163
3	R0	{2.57891, 12.5971}	{2.47003, 5.55104}	{{1.76886, 1.17738}, {3., 12.8554}}	{21.2033, 56.9725}	inf	340.	21 236.5	{4926.27, 53 998.3}	6146.03	1.63752	newly	303.	22 145.3	{3261., 55 418.}	7208.68	1.66807	Reff	2.5648	0.563695	{0.14404, 1.08991}	78.0219	ar	90 302.	144 412.	{76 726., 207 179.}	67.6029
4	R0	{2.5856, 9.28508}	{2.16694, 9.15833}	{{1.02412, 2.11088}, {3., 12.8775}}	{29.0664, 33.0038}	inf	8.	16 051.3	{2975.16, 30 822.}	200 541.	3.13976	newly	20.	20 881.9	{1454., 47 893.}	104 310.	2.77406	Reff	2.58508	1.66745	{0.375647, 2.58026}	37.4889	ar	90 312.	121 780.	{60 682., 192 394.}	50.0906
5	R0	{2.4406, 12.1341}	{2.60207, 7.81015}	{{2.16206, 4.19565}, {3., 14.7481}}	{20.4302, 40.8076}	inf	184.	23 800.	{8460.41, 43 003.5}	12 834.8	2.00489	newly	170.	20 756.6	{6419., 44 479.}	12 109.7	1.96176	Reff	2.43279	0.824804	{0.312418, 1.83133}	66.0964	ar	88 597.	146 010.	{84 074., 237 593.}	69.4521
6	R0	{2.72155, 15.4094}	{2.12526, 3.94744}	{{1.1387, 2.65186}, {3., 4.96848}}	{31.6686, 74.3829}	inf	411.	14 969.2	{4687.63, 40 558.3}	3542.95	1.37182	newly	302.	17 694.1	{2936., 56 019.}	5758.97	1.54379	Reff	2.7028	0.402751	{0.0759254, 1.13869}	85.0988	ar	91 682.	151 987.	{68 401., 197 598.}	77.6121
7	R0	{2.63781, 14.3935}	{2.5028, 5.13105}	{{2.0011, 1.57693}, {3., 7.29086}}	{19.6728, 64.3516}	inf	448.	2436.76	{681.335, 2371.37}	446.995	0.508473	newly	307.	3274.19	{789., 4853.}	969.306	0.691777	Reff	2.61787	0.512313	{0.134421, 1.25659}	80.4301	ar	91 192.	181 962.	{168 127., 231 225.}	100.782
8	R0	{2.82391, 16.104}	{2.30833, 3.97215}	{{1.04805, 2.64596}, {3., 5.00194}}	{25.0158, 75.3344}	inf	560.	14 185.4	{8340.25, 31 833.5}	2433.11	1.27814	newly	385.	16 462.6	{6382., 40 998.}	4176.	1.46764	Reff	2.79954	0.380304	{0.0462983, 0.797401}	86.4155	ar	92 664.	150 449.	{76 583., 193 983.}	71.2616
9	R0	{2.80645, 14.6457}	{2.08746, 4.31656}	{{1.07747, 2.64603}, {3., 4.97656}}	{31.2745, 70.6654}	inf	226.	13 155.3	{3037.36, 28 665.7}	5721.18	1.57183	newly	148.	14 671.7	{2688., 40 850.}	9813.34	1.778	Reff	2.79573	0.379445	{0.0802599, 0.817608}	86.4277	ar	92 567.	156 761.	{90 715., 210 740.}	75.3368
10	R0	{1.86213, 5.4271}	{2.27327, 9.79527}	{{1.58192, 2.38948}, {3., 17.0869}}	{39.8188, 93.8562}	inf	32.	27 193.2	{5350.89, 54 438.}	84 878.8	2.78018	newly	74.	26 353.4	{4249., 62 429.}	35 513.5	2.35531	Reff	1.8607	1.38932	{0.530533, 2.22213}	33.2457	ar	75 362.	128 689.	{65 254., 177 245.}	77.1493
11	R0	{2.27349, 14.6338}	{2.5999, 5.28285}	{{1.9892, 1.65539}, {3., 7.34832}}	{24.6859, 63.8996}	inf	1278.	2865.83	{841.416, 2891.55}	147.84	0.215275	newly	843.	3405.03	{739., 4172.}	315.225	0.318122	Reff	2.22036	0.442143	{0.149488, 0.710498}	80.0868	ar	85 805.	169 342.	{149 803., 194 966.}	100.296
12	R0	{2.77261, 14.1894}	{1.89003, 4.08315}	{{0.0783026, 0.912179}, {3., 5.79094}}	{39.5412, 71.3551}	inf	206.	5293.09	{1110.13, 13 769.4}	2469.46	1.21927	newly	178.	6334.36	{892., 18 080.}	3458.63	1.29323	Reff	2.76385	0.32475	{0.0112937, 0.580023}	88.2501	ar	92 508.	167 887.	{155 687., 200 724.}	84.3137
13	R0	{2.65402, 19.3935}	{2.46146, 6.89424}	{{1.45531, 3.4204}, {3., 9.77189}}	{21.0735, 64.4508}	inf	1354.	2782.78	{425.388, 4456.4}	128.33	0.259945	newly	658.	3608.58	{260., 3758.}	467.195	0.469645	Reff	2.59651	0.538955	{0.0180855, 1.45302}	79.2431	ar	91 184.	184 099.	{171 849., 231 497.}	102.852
14	R0	{1.75297, 4.45354}	{2.46283, 7.0827}	{{2.16544, 4.31202}, {3., 9.89049}}	{46.1173, 63.6001}	inf	18.	23 151.1	{7802.44, 42 022.3}	128 517.	2.93209	newly	29.	23 683.	{6353., 51 645.}	81 565.4	2.73381	Reff	1.75187	1.04163	{0.178577, 2.18143}	53.124	ar	71 263.	154 774.	{76 455., 233 822.}	119.619
15	R0	{1.99928, 7.1501}	{2.56411, 7.11786}	{{2.1604, 4.20433}, {3., 14.7329}}	{32.7025, 42.7398}	inf	97.	23 154.5	{6685.96, 56 148.}	23 771.5	2.22961	newly	168.	24 627.3	{5293., 59 267.}	14 560.8	1.98727	Reff	1.99548	0.927337	{0.337635, 1.97536}	55.2244	ar	79 602.	153 712.	{94 885., 221 629.}	96.2253
16	R0	{2.67398, 10.3134}	{2.47863, 7.14494}	{{2.16287, 4.25796}, {3., 10.8984}}	{17.7908, 39.2201}	inf	44.	24 381.9	{7405.55, 56 676.4}	55 313.5	2.62781	newly	54.	25 475.3	{6806., 59 945.}	47 076.5	2.53288	Reff	2.67219	1.02283	{0.288978, 2.18645}	61.7233	ar	91 289.	160 668.	{92 195., 236 982.}	80.095
17	R0	{2.44634, 16.3226}	{2.34469, 6.4352}	{{1.43125, 3.39301}, {3., 9.86625}}	{26.1379, 60.5749}	inf	1288.	2756.35	{1224., 5533.94}	124.812	0.213548	newly	777.	4088.56	{760., 6058.}	437.091	0.388848	Reff	2.39443	0.512782	{0.160469, 0.992282}	79.0381	ar	88 710.	177 678.	{167 848., 192 061.}	100.291
18	R0	{2.02168, 10.1676}	{2.55429, 4.89565}	{{2.00312, 1.87261}, {3., 7.29364}}	{32.5023, 51.8505}	inf	354.	1627.39	{737.97, 3423.09}	360.775	0.559988	newly	351.	2793.8	{721., 5041.}	697.51	0.608364	Reff	2.00733	0.473493	{0.139797, 1.12081}	76.4809	ar	80 299.	177 933.	{164 135., 201 269.}	121.588
19	R0	{2.89969, 15.009}	{2.06626, 4.22397}	{{1.05385, 2.64569}, {3., 4.97197}}	{31.7864, 71.8571}	inf	224.	15 431.3	{3560.45, 50 106.9}	6790.5	1.6468	newly	179.	17 624.2	{6411., 52 892.}	9747.21	1.77353	Reff	2.88972	0.413931	{0.064293, 1.11963}	85.6757	ar	93 349.	158 635.	{91 034., 210 138.}	75.6492
20	R0	{2.68933, 7.34928}	{2.15991, 9.36835}	{{0.441149, 2.11085}, {3., 13.6112}}	{29.3471, 52.9586}	inf	3.	17 719.1	{6988.75, 34 859.2}	590 535.	3.66618	newly	7.	24 696.8	{5111., 81 199.}	352 712.	3.33893	Reff	2.68909	1.63977	{0.187097, 2.99997}	42.2515	ar	91 574.	120 249.	{51 894., 160 803.}	46.6261
21	R0	{2.15352, 9.28074}	{2.53943, 7.1009}	{{2.14631, 4.22477}, {3., 14.7347}}	{23.231, 39.806}	inf	131.	25 937.9	{7828.51, 56 391.6}	19 700.4	2.18631	newly	151.	27 535.7	{6980., 72 468.}	18 135.8	2.11245	Reff	2.14827	0.976376	{0.379587, 2.18722}	55.291	ar	83 845.	159 795.	{85 792., 239 601.}	94.7827
22	R0	{2.21953, 9.04387}	{1.98732, 6.09179}	{{0.839546, 2.17327}, {3., 10.0798}}	{37.8484, 39.0969}	inf	114.	26 777.4	{7668.51, 49 239.5}	23 389.	2.26635	newly	136.	34 302.3	{9176., 73 005.}	25 122.3	2.23717	Reff	2.21565	0.985741	{0.345222, 1.69755}	56.3125	ar	84 856.	141 872.	{88 625., 205 349.}	70.2179
23	R0	{2.21987, 14.5347}	{2.23915, 8.82968}	{{0.363894, 2.95466}, {3., 13.0448}}	{43.2754, 39.2511}	inf	1324.	5623.52	{2781.85, 2781.85}	324.737	0.412063	newly	822.	4450.48	{1204., 3830.}	441.421	0.4445	Reff	2.16759	0.372878	{0.0640176, 0.647875}	82.7976	ar	85 067.	184 588.	{175 422., 181 591.}	116.991
24	R0	{2.84268, 17.9659}	{1.92819, 9.20263}	{{0.420712, 2.67767}, {3., 13.8221}}	{36.8188, 48.7772}	inf	799.	5210.34	{3099.71, 8441.52}	552.108	0.682323	newly	439.	3826.95	{1287., 5013.}	771.743	0.75434	Reff	2.80769	0.349505	{0.0784655, 0.620692}	87.5518	ar	92 836.	193 330.	{183 634., 218 668.}	108.249
25	R0	{2.52706, 10.3679}	{2.17818, 4.60081}	{{0.0938567, 0.911411}, {3., 5.74316}}	{35.4222, 62.5691}	inf	97.	4760.06	{431.488, 9321.16}	4807.64	1.46684	newly	97.	5329.82	{383., 10 303.}	5394.99	1.45787	Reff	2.52279	0.328833	{0.0129227, 0.488792}	86.9655	ar	89 623.	152 493.	{117 884., 173 296.}	76.131
26	R0	{2.62835, 19.1088}	{2.53427, 7.43731}	{{1.45345, 3.3926}, {3., 9.88368}}	{20.2645, 61.0792}	inf	1699.	3504.5	{950.731, 3480.22}	144.316	0.218975	newly	814.	3448.79	{564., 4889.}	335.984	0.321642	Reff	2.55757	0.616336	{0.295257, 1.0333}	75.9015	ar	90 908.	189 234.	{177 350., 218 866.}	108.16
27	R0	{2.76831, 19.4155}	{2.56582, 4.8357}	{{2.01415, 1.14167}, {3., 7.63022}}	{17.023, 75.0936}	inf	2186.	1845.97	{424.677, 2069.41}	65.4555	0.334957	newly	1084.	3050.89	{472., 5115.}	216.942	0.322757	Reff	2.67358	0.516599	{0.175366, 1.21368}	80.6776	ar	92 175.	182 463.	{167 869., 202 098.}	97.953
28	R0	{2.96609, 16.8903}	{2.56508, 7.27984}	{{2.15708, 4.18722}, {3., 14.6824}}	{14.6401, 57.0973}	inf	591.	24 177.	{6854.24, 36 052.7}	3990.87	1.48825	newly	383.	27 552.2	{5764., 73 160.}	7093.79	1.66554	Reff	2.94132	0.994428	{0.353347, 2.12488}	66.1911	ar	94 024.	157 713.	{97 395., 236 594.}	71.5745
29	R0	{2.17046, 14.39}	{2.29527, 8.80885}	{{0.427897, 2.88656}, {3., 13.1714}}	{43.8108, 38.7849}	inf	1169.	5882.87	{3106.56, 3317.63}	403.24	0.516665	newly	743.	4435.47	{1339., 6917.}	496.968	0.54686	Reff	2.12193	0.394512	{0.0833333, 0.650904}	81.4079	ar	84 001.	183 768.	{174 358., 207 817.}	118.768
30	R0	{2.88772, 12.4647}	{2.11969, 9.26578}	{{1.12133, 2.11084}, {3., 12.9033}}	{29.1692, 33.1728}	inf	43.	14 901.1	{4086.46, 22 651.8}	34 553.8	2.41162	newly	59.	19 672.1	{3196., 49 872.}	33 242.5	2.29802	Reff	2.88584	1.58686	{0.30821, 2.11232}	45.536	ar	93 273.	120 970.	{53 514., 171 402.}	43.9274
31	R0	{2.46831, 8.14308}	{2.06796, 8.53085}	{{0.492009, 2.00079}, {3., 16.8631}}	{32.0557, 61.2676}	inf	13.	9633.75	{3853.46, 18 645.4}	74 005.8	2.75199	newly	16.	24 899.8	{2747., 79 792.}	155 523.	2.86743	Reff	2.46792	1.78758	{0.304049, 2.94403}	35.6782	ar	88 762.	82 668.4	{22 274., 108 121.}	30.307
32	R0	{1.93236, 7.38987}	{2.33454, 6.2065}	{{0.849295, 2.17235}, {3., 10.095}}	{43.8739, 44.1351}	inf	96.	28 727.8	{5337.97, 54 352.3}	29 824.8	2.32847	newly	158.	36 072.	{5236., 74 611.}	22 730.6	2.16076	Reff	1.92828	1.06436	{0.276533, 1.72224}	48.0588	ar	77 654.	135 052.	{65 602., 197 367.}	82.0272
33	R0	{2.82251, 17.6954}	{2.4284, 3.79304}	{{1.8075, 1.00222}, {3., 13.0419}}	{19.6228, 78.5648}	inf	586.	24 214.5	{4786.74, 54 196.4}	4036.86	1.44637	newly	325.	31 087.	{4681., 78 947.}	9467.71	1.76538	Reff	2.7977	0.634958	{0.232809, 1.36366}	77.3043	ar	92 745.	152 340.	{92 865., 254 764.}	73.3574
34	R0	{1.87083, 12.3994}	{2.28106, 8.20722}	{{0.410349, 2.87585}, {3., 12.9922}}	{54.6652, 37.9569}	inf	1336.	3966.22	{2643.04, 2643.04}	196.873	0.348617	newly	975.	3531.98	{1135., 3583.}	262.254	0.344208	Reff	1.81905	0.350217	{0.0661773, 0.466163}	80.7472	ar	76 282.	193 939.	{187 355., 191 993.}	154.24
35	R0	{2.95779, 16.3003}	{2.4057, 4.93197}	{{1.76199, 1.1082}, {3., 12.8075}}	{20.0356, 69.743}	inf	233.	23 048.8	{4837.5, 54 322.5}	9793.96	1.82972	newly	159.	27 207.7	{4904., 67 532.}	17 011.8	2.01527	Reff	2.94682	0.642584	{0.24668, 1.36642}	78.194	ar	93 873.	157 208.	{97 865., 238 027.}	71.349
36	R0	{2.18718, 14.261}	{2.10361, 4.13483}	{{0.0724171, 0.927331}, {3., 5.74111}}	{44.428, 71.2431}	inf	1258.	4145.65	{948.854, 5057.78}	242.725	0.412262	newly	857.	4717.63	{713., 7192.}	458.708	0.55364	Reff	2.13862	0.31427	{0.0169994, 0.457741}	85.3051	ar	84 379.	161 338.	{89 529., 182 208.}	98.9319
37	R0	{2.44912, 9.31118}	{2.39441, 10.5034}	{{1.57974, 7.64907}, {3., 17.1191}}	{26.5263, 38.4111}	inf	41.	25 704.	{6565.83, 39 140.8}	62 592.6	2.6696	newly	54.	21 289.9	{4580., 31 594.}	39 325.8	2.42347	Reff	2.44728	1.28582	{0.469263, 2.22035}	49.266	ar	88 442.	131 296.	{89 181., 173 081.}	52.8071
38	R0	{2.05949, 5.72514}	{2.31009, 10.016}	{{1.55376, 6.56118}, {3., 17.1098}}	{34.9888, 86.2396}	inf	11.	25 134.	{7697.33, 54 300.9}	228 391.	3.25438	newly	26.	23 933.6	{5461., 36 656.}	91 952.3	2.81069	Reff	2.05842	1.41054	{0.524937, 2.2223}	38.2037	ar	81 587.	131 202.	{88 675., 179 576.}	61.673
39	R0	{1.75505, 8.52404}	{2.47549, 4.8437}	{{1.2114, 1.61141}, {3., 7.33266}}	{49.137, 44.1746}	inf	489.	2086.78	{792.786, 3185.76}	336.475	0.479254	newly	574.	3451.09	{719., 6546.}	512.197	0.472816	Reff	1.73539	0.503467	{0.136872, 1.06666}	72.8748	ar	70 925.	190 388.	{178 079., 211 150.}	168.435
40	R0	{2.24724, 13.7861}	{2.09663, 4.0207}	{{0.0504924, 0.94058}, {3., 5.71362}}	{45.2887, 71.0802}	inf	1281.	4380.53	{752.922, 10 420.1}	259.742	0.458376	newly	906.	4755.8	{523., 10 714.}	441.199	0.585708	Reff	2.19627	0.296727	{0.00725873, 0.457595}	86.4895	ar	85 257.	153 729.	{62 817., 177 045.}	91.1916
41	R0	{1.77635, 8.62406}	{2.20574, 4.59171}	{{1.08215, 2.65297}, {3., 4.9733}}	{53.2431, 50.18}	inf	634.	14 086.5	{8696.55, 27 066.2}	2121.85	1.23234	newly	682.	15 180.3	{5909., 35 324.}	2125.84	1.20138	Reff	1.75212	0.36539	{0.0407898, 0.845176}	79.1458	ar	72 941.	153 216.	{70 852., 198 363.}	114.218
42	R0	{1.80397, 5.4932}	{2.27376, 9.76104}	{{1.34698, 1.992}, {3., 17.1313}}	{44.9455, 93.2633}	inf	36.	27 423.2	{6301.07, 71 436.4}	76 075.6	2.73358	newly	83.	25 039.8	{5892., 47 783.}	30 068.5	2.29852	Reff	1.80242	1.30954	{0.240142, 2.22214}	39.9754	ar	73 232.	136 804.	{78 642., 194 735.}	88.644
43	R0	{2.83096, 16.7077}	{2.39524, 6.53454}	{{1.46661, 3.41649}, {3., 9.9185}}	{21.3624, 60.8891}	inf	491.	3041.97	{373.21, 6431.36}	527.671	0.575886	newly	311.	4140.68	{227., 8878.}	1239.61	0.746718	Reff	2.80905	0.600771	{0.0591548, 1.46425}	78.613	ar	92 740.	191 963.	{177 196., 242 046.}	106.991
44	R0	{2.73678, 11.6021}	{2.15732, 6.27036}	{{0.847408, 2.17251}, {3., 10.116}}	{30.4062, 48.6075}	inf	128.	25 406.9	{8767.76, 42 390.9}	19 749.1	2.19533	newly	125.	32 149.9	{8458., 76 580.}	25 619.9	2.24724	Reff	2.73147	1.00172	{0.350795, 1.61979}	63.3265	ar	92 003.	135 447.	{94 812., 190 866.}	49.8099
45	R0	{2.14235, 6.98601}	{2.52147, 6.65461}	{{2.16921, 4.15126}, {3., 9.99265}}	{22.4181, 45.9497}	inf	49.	22 945.7	{7462.75, 50 405.9}	46 727.9	2.52184	newly	74.	27 879.4	{6041., 64 058.}	37 575.1	2.36945	Reff	2.14079	0.959853	{0.223706, 2.18229}	56.263	ar	82 974.	151 936.	{81 654., 226 831.}	87.3527
46	R0	{2.23409, 9.21289}	{2.56297, 6.64479}	{{2.15987, 4.36495}, {3., 9.78061}}	{19.3919, 41.5594}	inf	58.	20 317.6	{3381.17, 36 867.2}	34 930.3	2.3839	newly	78.	25 493.9	{4333., 60 698.}	32 584.5	2.28476	Reff	2.23183	1.06574	{0.432526, 2.1853}	52.2481	ar	85 163.	155 268.	{90 281., 234 656.}	86.3574
47	R0	{2.12258, 8.15265}	{2.30852, 10.7378}	{{1.58427, 7.62257}, {3., 17.107}}	{32.0045, 48.309}	inf	73.	24 166.8	{5394.86, 49 080.6}	33 005.1	2.39501	newly	100.	22 544.4	{2759., 34 680.}	22 444.4	2.13599	Reff	2.11978	1.3112	{0.398402, 2.2202}	41.6884	ar	82 690.	127 322.	{77 864., 172 521.}	57.9032
48	R0	{2.71771, 10.4636}	{2.34038, 10.3223}	{{1.58216, 6.05366}, {3., 17.132}}	{24.0084, 38.3775}	inf	20.	29 789.5	{8290.59, 75 122.}	148 848.	3.0415	newly	23.	25 478.2	{6685., 46 607.}	110 675.	2.88758	Reff	2.71654	1.30901	{0.320117, 2.22211}	51.8383	ar	91 716.	140 605.	{94 160., 180 461.}	55.7442
49	R0	{2.44369, 6.66477}	{1.88893, 8.31214}	{{0.425632, 2.00079}, {3., 14.8806}}	{38.9602, 75.2792}	inf	7.	8226.06	{1881.93, 15 830.3}	117 415.	2.89013	newly	14.	19 711.1	{1897., 60 823.}	140 693.	2.76367	Reff	2.44335	1.70225	{0.302956, 2.95614}	39.2376	ar	88 827.	80 006.	{18 061., 109 266.}	38.3685
50	R0	{2.77383, 17.9775}	{2.22394, 6.63892}	{{1.43339, 3.41212}, {3., 9.9343}}	{28.1411, 63.0709}	inf	1401.	2517.75	{796.586, 5019.05}	89.4646	0.179	newly	799.	3835.63	{505., 6555.}	389.773	0.368598	Reff	2.71353	0.501963	{0.00856484, 1.00666}	81.5015	ar	92 334.	178 858.	{169 951., 203 799.}	93.708
